# Metacognition and Mindfulness: the Role of Fringe Consciousness

**DOI:** 10.1007/s12671-016-0494-z

**Published:** 2016-02-22

**Authors:** Elisabeth Norman

**Affiliations:** Department of Psychosocial Science, Faculty of Psychology, University of Bergen, Christiesgate 12, 5015 Bergen, Norway

**Keywords:** Mindfulness, Metacognition, Metacognitive feelings, Experience-based feelings, Fringe consciousness, Consciousness

## Abstract

The involvement of metacognition in mindfulness is already acknowledged in recent mindfulness models. The focus of the current paper is on how mindfulness may be seen to involve a particular subcategory of metacognitive feeling referred to as *fringe consciousness*. Fringe feelings are in themselves consciously experienced but have been demonstrated to reflect nonconscious context information and are assumed to play a functional role in metacognitive monitoring and behavioral control. I first address ways in which metaexperiences during mindfulness may be seen as a variety of fringe consciousness. I then turn to how mindfulness practice may change a person’s attitude to fringe feelings, which in turn may influence the ease with which currently unconscious cognitive content may be retrieved. Finally, I specifically discuss how *feelings of novelty*, described by many as characteristic of a mindful state, may be understood within the fringe consciousness framework. I propose that fringe consciousness may be a useful framework for understanding the relationship between cognition and certain forms of subjective feelings during mindfulness.

## Introduction

Over the last years, there has been an increased interest in the relationship between mindfulness and metacognition (e.g., Jankowski and Holas 2014; Shapiro, Carlson, Astin, and Freedman 2006), which can broadly be defined as cognition about one’s own cognition (Koriat 2007; Metcalfe 2000; Dunlosky and Metcalfe 2009; Nelson 1990). The relevance of metacognition to mindfulness can be illustrated by the fact that heightened attention and an open orientation to one’s own mental events, which are characteristic of mindful states (e.g., Bishop et al. 2004), both require that the person *monitors* and *controls* aspects of their ongoing cognitive activity, which are the two core functional mechanisms of metacognition (Nelson 1990).

Shapiro et al (2006) specifically linked mindfulness to metacognition, arguing that the process of mindfully attending with openness and nonjudgementalness leads to what is referred to as *reperceiving*. This involves a shift in perspective, where thoughts, feelings, and sensations that were previously “subject” now become “object” (Shapiro et al. 2006), in the sense that they are experienced more independently of one’s expectations, experience, or attitudes. This involves a more flexible attitude to one’s experiences. The contrasting state of mind would be *mindlessness*, during which the individual is highly context-dependent, ignorant to novel aspects of their environment, and overreliant on learned schemas and scripts (Langer 1992).

In Jankowski and Holas’ (2014) “metacognitive model of mindfulness,” different levels of metacognition in mindfulness, as well as different subcomponents of metacognition and the relationship between them, are specified and discussed. In addition to the distinction between an *object level* and a *meta*-*level* (Nelson 1990), suggested by Shapiro et al. (2006) to be central to understanding mindfulness, they suggest that metacognition during mindfulness can also take place at a *meta*-*meta level*. At the meta-level, metacognitive thoughts or experiences relate to raw, first-level experience or *qualia*—for example, “my breathing is shallow.” At the meta-meta-level, the object of metacognition is in itself metacognitive, e.g., “I am just thinking of the qualities of my breathing.” The model represents a step toward a more detailed understanding of the (meta)cognitive mechanisms involved in mindfulness, and also a closer integration of different traditions within mindfulness research and practice (Brown and Ryan 2003; Kabat-Zinn 2003). Furthermore, the model is compatible with recent attempts to draw parallels between research on metacognition and metaemotion—i.e., emotions about emotions (Norman and Furnes 2016).

Interestingly, Jankowski and Holas (2014) suggested that even though metacognitive experiences during mindfulness are always conscious, they may sometimes result from monitoring and judging processes that may be implicit and unconscious. There are clear similarities between this form of experience-based metacognitive feelings and *fringe consciousness*. Even though Jankowski and Holas (2014) briefly discussed fringe consciousness as a metacognitive phenomenon, they did not specifically address how it may be involved in mindfulness. This is the question I raise in this paper. I propose three different forms the involvement of fringe consciousness in mindfulness may take. In my view, more knowledge about metacognitive experiences during mindfulness, and how they might relate to aspects of cognitive processing, is important in order to obtain a better insight into the mechanisms involved in mindfulness and its effects.

## Fringe Consciousness as a Concept Relevant to Mindfulness

Fringe consciousness was first described by James (1890). He referred to the substantive, lingering, and articulate aspects of consciousness as “the nucleus,” and the transitive, fleeting, and inarticulate aspects as “the fringe.” Common examples of fringe feelings are *tip*-*of*-*the*-*tongue* experiences, *feelings of rightness*/*wrongness*, *feelings of familiarity*/*novelty*, and *feelings of knowing* (Mangan 2001, 2014, 2015). What these phenomena have in common is the presence of a subjectively experienced feeling in the absence of current conscious awareness of the information-processing antecedents of that feeling (Mangan 2001; Norman 2002, 2010; Norman Price and Duff 2010). Fringe consciousness can be seen as a summary representation of information that in itself is currently inaccessible to consciousness, but nevertheless relevant to the current contents of focal awareness (Mangan 2001). Importantly, the absence of such access is perceived as anomalous or surprising because it occurs in a situation in which the person would normally expect there to be conscious access to the information in question (Price and Norman 2008). For instance, because a feeling of familiarity occurring at the sight of a person would in most cases be associated with conscious recollection of the person’s identity, the lack of such information may be perceived as anomalous.

Fringe consciousness is sometimes described as vague. One way of understanding vagueness is in terms of lack of clarity of the subjective experience itself (Mangan 2001), or the difficulties in verbalizing it (Price 2002). However, not all fringe feelings are vague in this sense—for instance, a tip-of-the-tongue experience may be both strong and vivid (Price 2002), and could also be introspected upon (Norman et al. 2010). However, fringe feelings could still be seen as vague in the sense that they do not themselves contain the information they represent (Galin 1994; Norman et al. 2010; Price 2002). Importantly, fringe consciousness is suggested to have a retrieval function (Mangan 2001). This implies that attempts to direct attention to fringe feelings, or hold attention on them, may help the individual redirect attention to the information it represents (Norman 2002).

Interestingly, Jankowski and Holas pointed to the possible involvement of fringe conscious experience in mindfulness, although not discussing the question in depth. First, they pointed to the possibility that mindfulness practice may increase a person’s sensitivity to “vague, transient and elusive meta-feelings” like the tip-of-the-tongue experience (Jankowski and Holas 2014, p. 70). Furthermore, they mentioned *feelings of novelty* as an example of feelings that occur during mindfulness practice.

## A Proposed Role of Fringe Consciousness in Mindfulness

In order to more specifically address how fringe consciousness may be involved in mindfulness, I propose three assumptions, namely that (1) metaexperiences during mindfulness may have the quality of fringe consciousness, (2) mindfulness practice may change the person’s attitude to fringe feelings, and (3) feelings of novelty during mindfulness may be understood as a variety of fringe consciousness.

### Metaexperiences During Mindfulness May Have the Quality of Fringe Consciousness

There are several reasons for assuming that metaexperiences during mindfulness may have the quality of fringe consciousness.

Fringe consciousness is a form of metacognitive experience that provides an “overtone” to, but is still separate from, the contents of focal attention (Mangan 2014). For example, it has been argued that aesthetic experiences are characterized by feelings of rightness (Mangan 2015), but where the person lacks full conscious access to what particular aspect of the stimulus situation gave rise to the subjective feeling state. Through coloring the primary sensory content, fringe feelings may also alter the experiential quality of the primary sensory content to which they relate—just like it has been suggested that metacognition during mindfulness may change the person’s first-order experience (Jankowski and Holas 2014).

Second, both fringe consciousness and mindful states have an elusive quality that renders them difficult to introspect upon. As pointed out by Block-Lerner, Salters-Pedneault, and Tull (2005), because mindfulness and acceptance by their very nature relate to the present moment, attempts to capture these processes are limited. Similarly, fringe consciousness is often described as elusive. For example, Baars (1988) referred to the tip-of-the-tongue state as a “fleeting conscious event” (p. 68), and Mangan (1993) described fringe feelings as “slippery, elusive, ungraspable” (p. 95). However, even though both forms of experience are described as elusive, it has been suggested that different fringe conscious experience differ in terms of their degree of elusiveness. Whereas some fringe feelings are short-lived and difficult to introspect upon, others are more long-lasting and can be experienced more strongly (Norman 2002). Similar assumptions could be made about mindful states.

If metacognitive experiences during mindfulness sometimes take the form of fringe consciousness, this may also help resolve the apparent tension between, on the one hand, the assumption that mindfulness involves metacognitive experiences (Jankowski and Holas 2014), and the claim that mindfulness is characterized by so-called *phenomenological reduction* (Brown and Cordon 2009). According to Brown and Cordon, the experiential quality of mindfulness is similar to the Husserlian concept *second mode of processing*. This is in contrast to a *natural attitude* in which sensory input is embedded in the automatically occurring evaluations or judgments about them, because “…not even a single, normal sensory perception is supposed to correspond to things existing in themselves” (Husserl 2008 p. 150). This view of mindfulness as involving unfiltered and receptive perception may seem at odds with the metacognitive perspective of mindfulness. In my opinion, fringe consciousness may provide a way of understanding how these two viewpoints may nevertheless be compatible: if every focal content is associated with fringe consciousness, then this would apply no less to mindful states than to other psychological states. Possible differences between them may instead relate to the extent to which the person pays attention to fringe consciousness, or the quality of fringe consciousness during mindful versus nonmindful states.

Based on the above arguments, I would hypothesize that mindfulness may be characterized by fringe consciousness. If mindful experience sometimes has the quality of fringe consciousness, this could also imply that mindfulness training may increase the frequency with which fringe consciousness is experienced during mindfulness, and could also have an impact on the frequency and quality of fringe experiences in other situations. It could also be hypothesized that the frequency with which one experiences mindful states in general, and fringe conscious states in particular, is influenced by certain individual difference variables. One candidate is openness to experience, which has already shown to be related to both mindfulness and fringe consciousness. Baer, Smith, Hopkins, Krietemeyer, and Toney (2006) reported a positive relationship between scores on the Mindful Attention Awareness Scale and “openness to experience.” Similarly, Norman, Price, and Duff (2006) found that individuals who scored higher on the NEO-PI Openness subscale “openness to feelings” showed more learning and more fringe awareness of acquired knowledge in an implicit learning task. Future studies may want to look at how personality variables like this may predict the frequency and intensity of fringe feelings during mindfulness.

### Mindfulness Practice May Change the Person’s Attitude to Fringe Feelings

Brown and Cordon (2009) are among those who have suggested that mindfulness may lead to a higher sensitivity to psychological or somatic signals, which in turn make the person more in control of these signals. This is also compatible with studies showing that mindfulness is related to increased levels of interoceptive awareness (e.g., Farb, Segal, and Anderson 2012). For example, it has been found that the introspective accuracy of experienced meditation practitioners, assessed in terms of tactile sensitivity for different body regions during a “body-scanning” meditation, was significantly higher than that of novices (Fox, Zakarauskas, Dixon, Ellamil, Thompson, and Christoff 2012). A plausible hypothesis, that is also compatible with claims made by Jankowski and Holas (2014), is that mindfulness training may also increase the person’s sensitivity to subtle, fringe feelings.

In mindfulness meditation, the goal of becoming able to “act without acting” (Jankowski and Holas 2014, p. 69) would in itself imply that the person has introspective access to his/her ongoing experience, keeps a decentered attitude toward this experience, and accepts whichever experiences may arise. This is clearly compatible with the view that sensitivity to fringe consciousness would be associated with a higher degree of conscious control over one’s feelings (Norman et al. 2006). However, it may seem at odds with fringe conscious experiences being described as vague in the sense of fringe feelings being perceived as lacking the information they represent (Norman et al. 2010; Price 2002). According to Price and Norman (2008), the felt gap between the purpose of a fringe feeling and the absence of the information that the feeling signals, would be perceived as anomalous or unexpected. For example, one may expect feelings of knowing occurring in memory situations to be accompanied by the searched-for knowledge, and feelings of familiarity for a person’s face to be accompanied by immediate conscious recollection of their identity (Norman et al. 2010). However, the mere idea of a feeling being experienced as *unexpected* implies that there is something one should *expect* from certain categories of feelings, which seems incompatible with the nonjudgmental attitude that is sometimes formulated as a goal in mindfulness meditation. For instance, acceptance may imply letting go of the struggle to control one’s experience (Block-Lerner et al. 2005). One hypothesis is therefore that the process of developing a more decentered attitude to one’s feelings also involves vagueness being experienced somewhat differently. For example, to a mindful person, a feeling of familiarity may be less likely to be perceived as unexpected or anomalous. Instead, the person may accept that certain feelings are not accompanied by the information they point to. In other words, to the extent that mindfulness practice may change a person’s attitude to fringe feelings, one hypothesis is that this change is related to the experience of vagueness.

A goal of mindfulness in psychotherapy may be to help the person become aware of previously inaccessible knowledge. According to Brown and Cordon (2009), “… a more sensitive awareness can lead to the uncovering of (perhaps challenging) psychological or somatic realities that can be given focused attention as a means to investigate, more fully process, and thereby better regulate and transcend them.” (p. 65). The question is then how a more decentered attitude, in which feelings are less likely to be experienced as unexpected or anomalous, would impact on the process of gaining access to previously unconscious information. One possibility is that a person with a decentered attitude to fringe feelings will be less likely to attempt retrieving the sought-after information. For example, even though the person has a strong feeling of familiarity at the sight of someone, they may choose to not consciously try to remember the person’s identity. The question is then whether this would reduce or increase the likelihood of gaining access to information that was previously inaccessible. Based on what is known about passivity effects in nonconscious perception (Price 2001), one may predict a passive attitude to be beneficial under certain circumstances. This is also compatible with Mangan’s (2001) claim that fringe feelings are, by nature, impossible to “figuratively grasp or squeeze” (Sect. 3.7), and that attempts to attend to them will therefore never fully succeed. From this perspective, a mindful attitude characterized by detachment may in fact facilitate retrieval of relevant nonconscious information rather than hinder it. However, the likelihood of gaining access to previously unconscious information may also be indicated by the relative salience of the feeling itself (Norman 2002). If potential accessibility is high, as in the transition between different thoughts in a line of thoughts, any fringe feelings will have a *fleeting* quality because attempts to direct attention to them will bring another focal content into focus. In contrast, when the information in question is highly inaccessible, as in implicit learning or memory, the resulting feeling will have a *frozen* quality and be possible to focally attend.

It should be added that even if mindful states may be characterized by fringe feelings, and even if mindfulness would increase people’s sensitivity to and awareness of them, I am not arguing that all metacognitive experiences involved in mindfulness would qualify as being fringe conscious, neither am I attempting to specify the exact conditions under which such feelings would occur—these questions would require detailed empirical investigation. In addition, the possible effect of mindfulness on people’s attitude to fringe feelings may of course not only be limited to fringe feelings during mindfulness, but also apply to fringe conscious experiences in other contexts.

### Mindfulness May Be Associated with Fringe Feelings of Novelty

A particular form of metaexperience that according to Jankowski and Holas (2014) is characteristic of mindfulness, is the *feeling of novelty*, i.e., that the perception of situations and objects that the person is already familiar with, nevertheless is experienced in a new and different way. It is also referred to as *beginner*’*s mind* (Bishop et al. 2004), or feelings of discovery or surprise (Mason and Hargreaves 2001).

Jankowski and Holas (2014) explained feelings of novelty within the framework of construal level theory (Trope and Liberman 2010), according to which the felt psychological distance to an object or a person has implications for the level of abstraction with which the object or person is represented, with increased distance being associated with more global representations. If a mindful attempt of “being here and now” (Jankowski and Holas, p. 70) reduces psychological distance to objects in one’s imediate environment, this may involve more local and detailed perceptual processing. Jankowski and Holas further argued that this type of processing reduces effects of expectation on perception, which further increases the likelihood that stimuli and events are perceived as novel.

From a fringe consciousness perspective, one may make some additional predictions concerning the nature of feelings of novelty and the circumstances under which they occur during mindfulness.

First, feelings of novelty could be seen as not only occurring when there is a shift from global to local perceptual processing, but also when stimuli in focal awareness activate different associations than before, i.e., other nonconscious knowledge representations. This could for example be the result of new associations being learned during mindfulness practice. As an illustration, in a qualitative study of mindfulness-based stress reduction, a patient with a history of depression reported feelings of discovery/surprise, and an opening of the mind (Mason and Hargreaves 2001) related to the realization that “what goes through your mind are just mental phenomena, they are just thoughts, not necessarily truths” (p. 204). In cases like these, it may be hypothesized that what gives rise to feelings of novelty (or discovery/surprise) may be that stimuli previously associated with rumination and worry have become associated with, e.g., relaxation and acceptance.

Second, feelings of novelty could be seen as signaling a change in the overall quality of cognitive representations. According to Winkielman, Ziembowicz, and Nowak (2015), fringe consciousness may be seen as reflecting what they refer to as "global processing experiences,” i.e., the overall properties of knowledge representations at the neural level. Interestingly, Winkielman and Nowak (2005) specifically referred to feelings of novelty as a fast feedback signal that informs the person about how current contents of focal awareness relates to knowledge representations. From this perspective, feelings of novelty may even be regarded as the conscious correlate of the characteristic patterns of cortical activation that have been documented to result from mindfulness training (see, e.g., Farb et al. 2007; Farb et al. 2012; Hölzel et al. 2011).

It should be noted that even though “feelings of novelty” addressed in mindfulness research most often concern familiar events or objects which are perceived in a different way than before, feelings of novelty may also occur when the person directs attention to parts of the environment that have been encountered in the past, but previously not been attended to in much detail. One example is the experience of the sensation underneath one’s feet.
